# Vulture Exclusion Halves Large Carcass Decomposition Rates and Doubles Fly Abundance

**DOI:** 10.1002/ece3.71408

**Published:** 2025-05-08

**Authors:** Julia Grootaers, Greta Hernández Campos, Violeta Marie Montenegro, Rosio Vega Quispe, Sarah Wicks, Sara Campos Landázuri, Eduardo Fabrizio Tubelli, Francisco Vega‐Reyes, Enzo Basso, Andrew Whitworth, Andrew Young, Christopher Beirne

**Affiliations:** ^1^ Centre for Ecology and Conservation, College of Life and Environmental Sciences University of Exeter Cornwall UK; ^2^ Asociación Conservación Osa Puerto Jiménez Costa Rica; ^3^ Department of Environmental Systems Science ETH Zürich Switzerland; ^4^ Bird Ecology Lab, Instituto de Ciencias Marinas y Limnológicas Universidad Austral de Chile Valdivia Chile

**Keywords:** carrion decomposition, ecosystem services, invertebrate, neotropics, scavenger, vulture

## Abstract

Carcass consumption by scavengers plays a critical role in wildlife and human health by providing services that maintain ecosystem functioning and potentially mitigate disease spreading. Vultures are particularly efficient scavengers, but their populations have sharply declined in Europe, Asia and Africa, raising concerns about similar declines in the comparatively less studied species of the Americas. While the effects of vulture absence on other vertebrate scavengers have been examined, the impact on invertebrate scavengers and their role in carrion decomposition remains unexplored. To determine the effects of vulture decline, specifically neotropical cathartid vultures, we experimentally excluded this functional group from domestic pig carcasses ( *Sus scrofa* ) in Costa Rica, under different habitat conditions (grassland and forest) and across seasons with the aim to assess the impact of vulture exclusion on carrion decomposition and insect abundance. Vulture exclusion halved carcass decomposition rates relative to control carcasses without exclusion. Accordingly, vulture abundance at control carcasses was positively correlated with carcass decomposition rate. Vulture exclusion doubled fly abundance at carcasses relative to controls but did not significantly impact dung beetle abundance at carcasses. These findings suggest that neotropical vultures are instrumental in rapid carrion decomposition, a service that invertebrates alone cannot fully compensate for, underscoring the potential ecological and public health risks associated with neotropical vulture declines and increased flies at carrion sites. Further research is needed to understand the broader implications of vulture loss on ecosystem services and zoonotic disease transmission in the Neotropics.

## Graphical abstract

We experimentally excluded vultures from pig carcasses ( *Sus scrofa* ) in Costa Rica, under different habitats and across seasons with the aim to assess the impact of vulture population decline on carrion decomposition and insect abundance. Vulture exclusion halved carcass decomposition rates and doubled fly abundance, while dung beetle abundance remained unaffected compared to control carcasses without exclusion. Our results indicate that neotropical vultures are instrumental in rapid carrion decomposition, a service which invertebrates alone cannot fully compensate for, underscoring the ecological and public health risks associated with potential neotropical vulture declines.

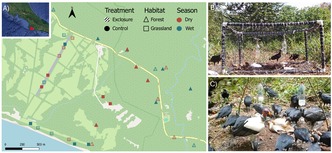

## RESUMEN

El consumo de animales en descomposición por parte de los carroñeros desempeña un rol fundamental en la salud de los ecosistemas, ya que proporcionan servicios que pueden mantener el funcionamiento del ecosistema y potencialmente mitigar la propagación de enfermedades. Los buitres son carroñeros particularmente eficientes en la eliminación de animales en descomposición del medio ambiente. Sin embargo, sus poblaciones han disminuido drásticamente en Europa, Asia y África. Esta situación ha generado una preocupación latente sobre potenciales declives similares en las especies de América, que han sido menos estudiadas en comparación. Aunque se han examinado los efectos que tiene la ausencia de los buitres en otros carroñeros vertebrados, el impacto en los carroñeros invertebrados y su papel en la descomposición de carroña permanece desconocido. Para determinar los efectos que podría tener el declive de las poblaciones de buitres catártidos neotropicales, realizamos experimentos de control y exclusión utilizando cadáveres de cerdos domésticos ( *Sus scrofa* ) en Costa Rica. Excluimos a los buitres para determinar el impacto en la tasa de descomposición de los cadáveres y en la abundancia de los carroñeros invertebrados. La exclusión de los buitres redujo a la mitad la tasa de descomposición de los cadáveres en comparación con los cadáveres de control sin exclusión. Asimismo, la abundancia de los buitres se correlacionó positivamente con la tasa de descomposición. La exclusión de los buitres duplicó la abundancia de moscas en las carroñas en comparación con los controles, pero no tuvo un impacto significativo en la abundancia de escarabajos peloteros. Estos resultados sugieren que los buitres neotropicales son fundamentales para acelerar la descomposición de la carroña, un servicio que los invertebrados por sí solos no pueden compensar completamente. Nuestros hallazgos resaltan los posibles riesgos ecológicos y de salud pública asociados con el declive de los buitres neotropicales y el aumento de moscas en los sitios de carroña. Se requiere información adicional para comprender de manera más amplia las implicaciones que podría tener la pérdida de buitres en los servicios ecosistémicos y la transmisión de enfermedades zoonóticas en los Neotrópicos.

## 1 Introduction

Human society and wellbeing are directly impacted by wildlife and ecosystem health. Seventy‐five percent of human diseases originate from animals (zoonoses) with transmission risk increasing due to heightened human‐wildlife interactions driven by climate and land use changes (Taylor et al. 2001; White and Razgour 2020; van den Heever et al. 2021). To optimise human, wildlife and ecosystem health, it is imperative we explore the implications of their interconnectedness (Ottinger et al. 2021; One Health Initiative 2022). An example of this is the benefit wildlife provides through ecosystem services such as carrion removal (DeVault et al. 2016). Carcasses host many microorganisms, some of which can be pathogenic to wildlife and humans (Janzen 1977; Houston 1979; Burkepile et al. 2006). By reducing the persistence of carrion within the ecosystem, scavengers may curb the spread of bacterial, viral or parasitic zoonoses (O'Bryan et al. 2018; Vicente and VerCauteren 2019; Newsome et al. 2021). Moreover, carcass removal by scavengers stabilises food webs (Wilson and Wolkovich 2011), helps nutrient cycling (DeVault et al. 2003; Newsome et al. 2021), and, in the case of avian scavengers, has been found to reduce the cost of carcass disposal services (Margalida and Colomer 2012). However, scavenger congregation at carcasses could also facilitate pathogen transmission by increasing contact between scavenger species, particularly if such species range over wide areas (Markandya et al. 2008; Ogada, Keesing, et al. 2012; Ogada, Torchin, et al. 2012). The dynamics of pathogen reduction through carcass consumption and the potential for zoonotic disease transmission has gained scientific attention over the past two decades but remains poorly understood (O'Bryan et al. 2018; Olea et al. 2019; Newsome et al. 2021). To reveal the full range of scavenger ecosystem services and the potential routes for zoonoses emergence, it is crucial to understand carcass decomposition processes and their agents in natural contexts.

Vultures are perhaps the best‐known vertebrate scavengers. As the most threatened avian guild, these obligate scavengers have experienced some of the steepest declines among vertebrates (Buechley and Şekercioğlu 2016; McClure et al. 2018; Santangeli et al. 2022). The International Union for the Conservation of Nature has listed 14 of the 16 Old World (family *Accipitridae*) and two of the seven New World species (family *Cathartidae*) as threatened or near threatened (IUCN 2024). Populations have dwindled across Asia, Africa and Europe due to poisoning, persecution and turbine or power line collisions (Green et al. 2004; Ogada, Keesing, et al. 2012; Ogada et al. 2016). In Asia, some species were brought back from near extinction by herculean conservation efforts after their population suffered a 99% decline (Pain et al. 2003; Prakash et al. 2003; Green et al. 2004). Both Old and New World vulture conservation efforts are now facing additional challenges due to the rise of online misinformation (“fake news”) about vulture attacks on livestock, which drives negative public perceptions of vultures (Lambertucci et al. 2021; Oliva‐Vidal, Hernández‐Matías, et al. 2022). Furthermore, stringent sanitation policies in an effort to reduce pathogen spread from infected livestock carcasses (e.g., bovine spongiform encephalopathy in Europe, see Margalida et al. 2010) can lead to rapid population collapse even in regions with high vulture abundance (Arrondo et al. 2018).

However, vulture carrion consumption may mitigate the spread of disease from carcasses to other wildlife, livestock and humans (Ogada, Keesing, et al. 2012; Ogada, Torchin, et al. 2012; Carlson et al. 2018; Plaza et al. 2020; van den Heever et al. 2021). Their specialised and highly acidic digestive system can destroy many of the pathogens found in carrion (e.g., Houston and Cooper 1975; Roggenbuck et al. 2014). For example, in India, the collapse of three vulture species due to accidental poisoning led to a surge in feral dogs filling their scavenger niche (Pain et al. 2003; Green et al. 2004; Oaks et al. 2004). This augmented the size of dog congregations at carcasses, facilitated rabies transmission and resulted in more rabies‐infected bites to humans and an increase in healthcare costs by an estimated $34 billion over 14 years (Markandya et al. 2008).

Several other services rendered by vultures benefit humans directly or maintain ecosystem functioning. Livestock carcass consumption by vultures obviates removal costs for farmers and is often the only viable way of carcass disposal in remote areas (Margalida and Colomer 2012; Santangeli et al. 2024). This in turn reduces the greenhouse gas emissions otherwise involved in transporting carcasses to incineration plants (Morales‐Reyes et al. 2015; Santangeli et al. 2024). Vultures also aid in the cycling of nutrients across the ecosystem, whether at smaller spatial scales within their home range or across continents from Europe to Africa or from North to South America during migrations (García‐Ripollés et al. 2010; Dodge et al. 2014; Alarcón and Lambertucci 2018). Consequently, their distribution of carcass biomass via scat deposition fertilizes soil across much larger distances than any other non‐avian scavenger (Ganz et al. 2012; Beasley et al. 2019). The global economic benefit of carcass removal and potential disease mitigation by vultures remains unquantified as we lack a full understanding of the services they provide (Carucci et al. 2022; but see Grilli et al. 2019).

Past research has focused heavily on the effect of vulture presence and absence on other scavenging vertebrates (e.g., Mateo‐Tomás et al. 2015; Morales‐Reyes et al. 2015) and on carrion consumption dynamics (e.g., Ogada, Keesing, et al. 2012; Ogada, Torchin, et al. 2012; Hill et al. 2018; Oliva‐Vidal, Sebastián‐González, et al. 2022). As apex scavengers, vultures are thought to carry out a top‐down regulatory function by outcompeting facultative scavengers (van den Heever et al. 2021). Hence, vulture decline has been associated with an increase in mesoscavengers such as foxes ( *Vulpes vulpes* ), feral dogs ( *Canis familiaris* ) and rats (genus *Rattus*) (Pain et al. 2003; Morales‐Reyes et al. 2017). This is concerning as vulture exclusion studies from both North America and Africa have demonstrated that despite higher numbers of facultative vertebrate scavengers at carrion, carcasses decompose more slowly under vulture exclusion (Ogada, Keesing, et al. 2012; Ogada, Torchin, et al. 2012; Hill et al. 2018). These studies confirmed that facultative vertebrate scavengers and invertebrates (although they did not explicitly investigate invertebrate activity at carrion) are unable to fully functionally replace the efficiency with which vultures remove carrion.

Similarly to facultative vertebrate scavengers, vulture decline could also increase carrion availability to insects and decomposer microorganisms (DeVault et al. 2004; Ray et al. 2014). Carcasses left un‐scavenged by vertebrates may be colonised by diverse insect communities including fly families such as *Calliphoridae*, *Sarcophagidae* and *Muscidae*, as well as carrion (family *Silphidae*) and dung beetles (family *Scarabaeidae*) (Muñoz‐Lozano et al. 2019; Griffiths et al. 2020). However, carcass decomposition activity by insects has traditionally been studied principally to estimate post‐mortem interval in forensic entomology (Griffiths et al. 2020). The effect vertebrates have on invertebrate scavengers and their ability to decompose carrion has largely been overlooked. The few studies available indicate that invertebrate activity can be an important mechanism of decomposition for small carcasses (e.g., DeVault et al. 2004; Sugiura and Hayashi 2018; Iyer et al. 2023 (preprint)). For example, burying beetles (genus *Nicrophorus*) were able to functionally compensate for low vertebrate density in two Japanese islands by removing more mouse carcasses than vertebrate scavengers (Sugiura and Hayashi 2018). Likewise, in the US, vertebrate scavengers removed just 35% of 300 rodent carcasses, suggesting that the majority of decomposition was a result of microbe and insect activity (DeVault et al. 2004). However, we lack studies on the effects of vertebrate scavenging on invertebrate activity and their abundance at larger carcasses. In the absence of vertebrate scavengers, large carcasses likely persist for longer, which may affect their use by invertebrates for consumption and reproduction, possibly leading to changes in their abundance (DeVault et al. 2004). Increased invertebrate abundance at carrion for longer periods of time may have important implications for zoonotic disease transmission risk as some species of dung beetles and flies are known disease vectors (e.g., Blackburn et al. 2014; Monyama et al. 2022; Patel et al. 2022).

To our knowledge, no experimental studies have been conducted on how vultures affect carrion decomposition and invertebrate scavenger activity in the Neotropics. Vulture research has almost exclusively concentrated on Old World species, exposing a prominent knowledge gap on scavenging by New World vultures, with neotropical species represented in just 7% of existing vulture literature (Santangeli et al. 2022). New World vultures face pressures similar to those of the Old World, prompting warnings of a potential population collapse in the Americas which is already manifest in species such as the critically endangered California Condor ( *Gymnogyps californianus* ) and the vulnerable Andean Condor ( *Vultur gryphus* ) (Birdlife International 2020a, 2020b; Bakker et al. 2023). Population declines could increase carrion availability to other scavengers, possibly slowing down carrion decomposition rates and increasing invertebrate abundance which, in turn, could have implications for healthy ecosystem functioning, zoonotic disease emergence and transmission (Ogada, Keesing, et al. 2012; Ogada, Torchin, et al. 2012; van den Heever et al. 2021; Carucci et al. 2022; Oliva‐Vidal, Sebastián‐González, et al. 2022). Aside from population declines, environmental variables such as habitat and season may also affect carrion consumption by vultures. For example, in northern Spain, carcass detection and the percentage of carcass consumed by griffon vultures ( *Gyps fulvus* ) decreased as vegetation cover increased (Oliva‐Vidal, Sebastián‐González, et al. 2022). Similar to their Old World counterparts, all New World species except the turkey vulture ( *Cathartes aura* ) rely solely on sight to locate carrion, meaning carcasses in grassland may be easier to detect than those concealed under a forest canopy (Houston 1985; Oliva‐Vidal, Sebastián‐González, et al. 2022). It has also been suggested that vultures fly more frequently in warmer weather in response to increased thermals, which could lead to higher carcass detection during the dry season compared to the wet season (e.g., Dodge et al. 2014).

Here, we experimentally excluded vultures from carcasses in the southern Pacific region of Costa Rica to test their impact on carrion decomposition rates and fly and dung beetle abundance. We contrasted the effects of vulture exclusion between forest and grassland habitats and between the wet and dry seasons via a balanced experimental design. Our predictions were that excluding vultures from carcasses will (1) decrease decomposition rate as seen in Africa and North America by Ogada, Keesing, et al. (2012), Ogada, Torchin, et al. (2012) and Hill et al. (2018), and (2) increase fly and dung beetle abundance due to longer carcass persistence times and decreased resource competition (DeVault et al. 2004). We also predicted that carcass decomposition rate at control sites would increase in (3) grassland compared to forest due to greater carcass visibility for vultures, as found by Oliva‐Vidal, Sebastián‐González, et al. (2022) in Europe, and in (4) the dry season compared to the wet season due to greater carcass detection probability as a result of increased vulture flight activity (Dodge et al. 2014). And lastly, to assess the functional redundancy of carrion removal by vultures in this ecosystem, we (5) examined the presence of other facultative vertebrate scavenger species at carcasses (Oliva‐Vidal, Sebastián‐González, et al. 2022).

## 2 Methods

### 2.1 Study Area

The Osa peninsula, in the southern Pacific region of Costa Rica, harbours the largest remaining tract of Pacific lowland wet forest in Central America (Holdridge 1967). The area is characterised by a tropical climate and its landscape comprises oil palm and pineapple plantations, cattle farms and secondary and primary forest (Gilbert et al. 2019). Temperatures range between 23.4°C and 28.8°C with seasonal rainfall between 3000 and 7000 mm year^−1^ (Taylor et al. 2015; Whitworth et al. 2018). Its wet season spans the months of June to November and its dry season from December through the end of May (Taylor et al. 2015).

**FIGURE 1:**
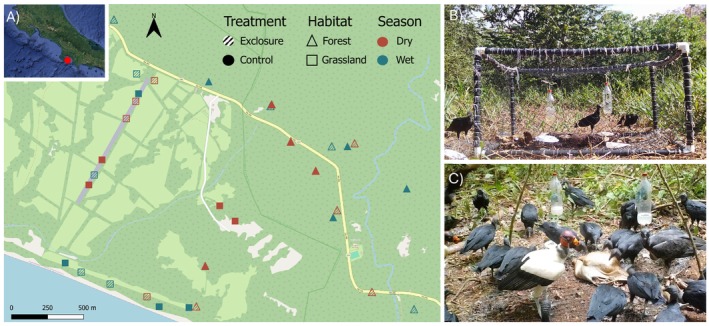
Study area within Costa Rica (inset) and the spatial arrangement of experimental sites on the Osa peninsula (A). Example exclusion site (B; in ‘grassland’ habitat) and example control site (C; in ‘forest’ habitat). The map was made using open‐source data in QGIS 3.34.11.

This study was conducted between September 2023 and June 2024 at the Osa Conservation Campus (8.40388° N, 83.336618° W; Figure 1A) situated within the Golfo Dulce Forest Reserve, which borders Corcovado National Park. Comprising 1330 ha of privately protected land, the Osa Conservation Campus boasts various habitat types, including old‐growth primary forest, secondary growth forest, secondary plantation forest and abandoned cattle pastures, which were cleared and actively farmed over 40 years ago (Whitworth et al. 2021).

### 2.2 Experimental Setup

The experiment consisted of pig carcasses (*Sus scrofa*; weight: 44–130 kg) deployed under two treatments: ‘exclusion’ – a pig carcass rendered inaccessible to large vertebrate scavengers by covering it with a cage (Figure 1B); and ‘control’ – where the carcass was left uncovered and accessible (Figure 1C). Pig carcasses were sourced from local farms supplying meat for human consumption. We deployed eight controls and eight treatments in secondary growth forest locations and eight controls and eight treatments in grassland locations, totalling 32 carcass deployments. The carcasses were deployed in eight ‘rounds’, with each round containing four carcasses placed at least 500 m from each other (as seen in Hill et al. 2018) in the following treatment and habitat combinations: Control in grassland, control in forest, exclusion in grassland and exclusion in forest. To account for seasonal variation, half of the experiment (four rounds) was conducted in the dry season and half (the other four rounds) in the wet season. The time between round deployments varied from 16 to 32 days, with one 60‐day wait period between the deployment of round four and five while the season changed from wet to dry. The sites chosen for the subsequent round were distanced at least 100 m from the previous round's sites.

### 2.3 Scavenger Exclusion and Monitoring

The exclusion cages set up around the carcasses were constructed using PVC pipes and netting measuring 0.8 m in height and 2 × 2 m in width and length (Figure 1B), with every corner of the cage staked into the ground. At control sites, the carcass was tied from both the rear and front legs to either a stake or a tree, impeding animals from dragging the carcass away. Carcasses were weighed from deployment day (day 0) for a minimum of 20 days or until they were completely consumed. The mean carcass persistence duration was 12 days (range 2–26 days). To monitor the integrity of the exclusion cages and determine vertebrate scavenger abundance, we deployed a camera trap (Browning model BTC‐5DCL) at each carcass, positioned approximately 1 m from the ground and two metres from the carcass. The cameras took pictures at 60 s intervals when motion was detected in the field of view, except for two which were set at 1 s intervals, from the start of the deployment until the end of the experiment. To account for the different sampling frequencies between camera deployments, only the first frame in a given minute was considered in subsequent analyses. Camera trap data were uploaded and individuals counted per frame and labelled to species level where possible using the Wildlife Insights platform (Wildlife Insights 2024).

We monitored invertebrate activity at each carcass using two methods: Pitfall traps and bottle traps (Barton and Evans 2017). Eight insect pitfall traps were placed around each carcass, four inner and four outer traps. The inner pitfall traps were positioned at 1 m distance from the centre of the carcass and equidistant from each other: Two at the front and two at the back of the carcass. At the exclusion sites, the inner pitfall traps were enclosed within the cage. It is unlikely that this impacted insect collection as the holes within the netting used measured 5 × 5 cm, allowing flies and beetles to easily pass through the mesh to the carcass. The outer pitfall traps were placed at 1 m distance from the inner pitfall traps following the same square configuration. Plastic plates held up with wooden skewers were used as pitfall roofs to prevent rain from entering the trap. Bottle traps were fashioned by cutting two 6 × 10 cm sized insect entrances spaced 4.5 cm apart at the top of 1.5 L empty plastic bottles. One hundred grams of pork bait in fine mesh was suspended inside each bottle via a string threaded through the lid. At each site, two bottle traps were hung 50 cm from the ground, one at either flank of the carcass. The bottles were attached to the roof netting within exclusion sites and from a line fastened between two upright sticks at control sites. Both the pitfall and bottle traps were half filled with a mixture of water and Florex non‐toxic washing‐up liquid (3 mL washing‐up liquid to every 1 L water) which kills and preserves invertebrates but is non‐toxic to vertebrates. A killing solution was necessary to prevent the insects from predating each other within the traps, which may skew abundance counts (Leather 2008).

Invertebrate collection occurred each day at dusk to minimise disturbance to vultures which are diurnal (Hill et al. 2018). Flies and dung beetles (only) were collected from both pitfall and bottle traps and stored in plastic Falcon tubes with 69% alcohol, after which they were counted at the Osa Conservation campus laboratory. We collected only dung beetles as carrion beetle distribution is primarily restricted to temperate regions, creating a vacant niche in the neotropics which seems to have been co‐opted by dung beetles (Peck 1985). Although three samples from single days at three different carcasses perished (out of a total of 17, 20 and 22 sampled days at each of these carcasses), our analysis remains unaffected because we calculated our abundance metrics adjusted to the number of successful sampling days.

### 2.4 Data Analysis

All statistical analyses were conducted using R Statistical Software version 4.4.2 (R Core Team 2024). For each response term of interest, we used either simple linear or mixed‐effects models depending on whether there was sufficient variation between deployment rounds for the mixed effects models to successfully converge. The latter were run with the ‘glmmTMB’ package (Brooks et al. 2017). For each model, described below, we used a model selection approach based on AIC comparisons of all possible model subsets using the MuMIn package (Symonds and Moussalli 2011; Bartoń 2023). We then calculated the Akaike weights (*w*_*i*_) to assess the weight of evidence in favour of each model (Burnham and Anderson 2004). We compare effect sizes to discuss the ecological significance of the statistically supported differences found and use pseudo‐*R* ^2^ (Nakagawa et al. 2013) to determine how well each of the best performing models describes the data.

#### 2.4.1 Experimental Vulture Exclusion Effect on Carcass Decomposition Rate

To test the effect of season, habitat and treatment on carcass decomposition rate (kg lost day^−1^), we first calculated the decomposition rate of each carcass (*n* = 32) by extracting the linear regression slope of carcass mass against days since deployment and then reversing its sign (rendering them positive estimates of mass loss per day rather than negative measures of mass gain per day). We used carcass mass from the first 10 days of carcass decomposition data for these calculations, or until the carcass was consumed (dropped below 20% of the original weight), whichever came first, as decomposition rates slowed substantially after these periods. To model the factors affecting decomposition rate we used a Gaussian distributed mixed‐effects model with deployment ‘round’ as a random intercept term to account for experiments occurring in the same round sharing similar environmental conditions. To control for any effect of variation in the initial masses of the carcasses we compared full models with and without initial carcass mass. Model fit did not improve with initial carcass mass and it was therefore excluded from model selection to reduce model complexity (i.e., models containing initial carcass mass scored weaker AICcs than models with initial carcass mass). Season (dry and wet), habitat (grassland and forest) and treatment (exclusion and control) were included as categorical fixed effect predictors. To assess the possibility of the effects of vulture exclusion varying between different habitat types and seasons, we included models which contained an interaction between habitat and treatment and between season and treatment.

To determine if vulture abundance at carcasses predicts carcass decomposition rate, all 16 carcasses at ‘control’ sites were analysed for vulture presence. We quantified an average metric of vulture abundance for each carcass using the camera trap images from the first 10 days of carcass deployment or until the carcass was consumed (whichever occurred first), mirroring the time course of our carcass decomposition rate calculations (see above). Since we could not distinguish between individual vultures across frames, we estimated their relative abundance by dividing the total number of vultures counted within the images collected at a given carcass by the total number of camera trapping days providing imagery from that site (O'Brien 2011). We then assessed the evidence for relative vulture abundance predicting carcass decomposition rate using a linear model with Gaussian distribution and comparing it to a null model without vulture abundance included.

#### 2.4.2 Experimental Vulture Exclusion Effect on Fly Abundance

To assess the fly abundance at a given carcass, we first summed the fly counts from both the bottle and pitfall traps at the site across all sampling days and then divided this by the number of sampling days (yielding a measure of flies per day (flies day^−1^); to control for variation in survey durations between deployments). Sampling occurred for a minimum of 20 days or until the carcass was fully decomposed (range 2–22 sampling days). We then analysed the effect of treatment, habitat and season on fly abundance using a negative binomial mixed‐effects model with deployment ‘round’ fitted as a random intercept term. To assess the possibility of the effects of vulture exclusion on fly abundance varying between different habitat types and seasons we included models with an interaction between habitat and treatment and between season and treatment. To assess if fly abundance predicted carcass decomposition rates in the absence of vultures, we then subsetted the data to only the exclusion sites and fitted a linear model with carcass decomposition rate as the response term and fly abundance as the predictor, before comparing its performance to that of a null model without fly abundance.

#### 2.4.3 Experimental Vulture Exclusion Effect on Dung Beetle Abundance

To assess dung beetle abundance at a given carcass, we first summed the dung beetle counts from both the bottle and pitfall traps at each site across all sampling days and then divided this by the number of sampling days (yielding a measure of dung beetles per day (dung beetles day^−1^); to control for variation in survey durations between deployments). We then analysed the effect of treatment, habitat and season on dung beetle abundance using a negative binomial mixed‐effects model with deployment ‘round’ fitted as a random intercept term. We included models with an interaction between habitat and treatment and between season and treatment. To assess if dung beetle abundance predicted carcass decomposition rates in the absence of vultures, we then subsetted the data to only the exclusion sites and fitted a linear model with carcass decomposition rate as the response term and dung beetle abundance as the predictor, before comparing its performance to that of a null model without dung beetle abundance.

## 3 Results

We confirmed that the exclusion cage worked effectively as camera trap footage showed that vultures did not feed on the pig carcass for 15 out of 16 exclusion replicates. In the single instance where a vulture did gain entry (day six of round eight within grassland habitat) we ended the deployment and only considered carcass decomposition and invertebrate abundance before the breach occurred. Vultures were recorded at all but one of the control carcasses. Of the 15 control carcasses visited by vultures, black vultures ( *Coragyps atratus* ) were the first obligate scavenger species detected to arrive at 53% of the carcasses, turkey vultures were first to arrive at 40% of the carcasses and king vultures ( *Sarcoramphus papa* ) were first to arrive at 7% of carcasses. Compared to vultures, other vertebrates were rarely detected at the carcass sites: Puma ( *Puma concolor* ; at 1/16 exclusions), ocelot ( *Leopardus pardalis* ; at 1/16 exclusions), coyote ( *Canis latrans* ; at 1/16 controls), agouti ( *Dasyprocta punctata* ; at 1/16 controls and 3/16 exclusions), white‐nosed coati ( *Nasua narica* ; at 1/16 controls and 2/16 exclusions), common opossum ( *Didelphis marsupialis* ; at 12/16 controls and 5/16 exclusions), spectacled caiman ( *Caiman crocodilus* ; at 1/16 exclusions) and collared peccary ( *Pecari tajacu* ; at 1/16 exclusions).

### 3.1 Experimental Vulture Exclusion Effect on Carcass Decomposition Rate

**FIGURE 2:**
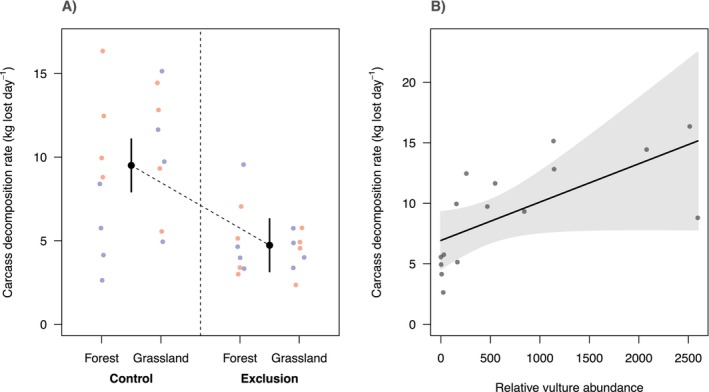
Effect of vulture exclusion on carcass decomposition rates (kg lost day^−1^) (A) and effect of relative vulture abundance on carcass decomposition rates at control sites (B). In panel (A), black points represent the predicted decomposition rates from the best supported model in Table 1, vertical bars represent 95% confidence interval for the fixed effect predictions, coloured dots represent the raw data (red = dry season; blue = wet season). In panel B, black line = predicted change in decomposition rate with increasing relative vulture abundance at a given control site; grey polygon represents the 95% confidence interval around the fixed effects; and grey dots represent the relative vulture abundance (control sites only).

There was strong support for treatment influencing carcass decomposition rates (Table 1: Figure 2A). The top model showed that carcasses available to vultures were consumed on average two times faster than those unavailable to vultures: Control carcasses lost an estimated 9.5 kg day^−1^ (95% confidence interval [CI] = 8.0–11.1), while exclusion carcasses lost an estimated 4.8 kg day^−1^ (CI = 2.65–6.91). The best supported model contained only the treatment effect (ΔAICc = −12.50 relative to the null model; Table 1) and explained 38.4% of the variation in carcass decomposition rate. The second top model supports a season and treatment interaction (ΔAICc = −12 relative to the null model; Table 1) and explained 48.2% of the variation in carcass decomposition rate. The effect of the exclusion treatment was dependent on season, as indicated by the interaction term (estimate = 3.83, CI = 0.08–7.74), suggesting that carcass decomposition rates are faster in the wet than the dry season under the exclusion treatment. There was no support for a habitat and treatment interaction predicting decomposition rates (Table 1; i.e., models containing this interaction scored weaker AICcs than the model with treatment alone). At control sites, there was strong support for relative vulture abundance influencing decomposition rates (ΔAICc = −6.62 relative to the null model), whereby the carcass decomposition rate increased from an average of 6.93 kg lost day^−1^ (CI = 4.6–9.3) at the lowest recorded vulture activity to 15.2 kg lost day^−1^ (CI = 7.8–22.5) at the highest recorded vulture activity (Figure 2B).

**TABLE 1:** Mixed‐effects model selection results explaining decomposition rate for 32 pig carcasses from which vultures were experimentally excluded or not (treatment) across two habitats (grassland and forest) and seasons (dry and wet).

Model	*k*	Log*L*	AICc	ΔAICc	*w*_*i*_
**Treatment + (1|Round)**	**4**	**−81.33**	**172.1**	**0.00**	**0.30**
Treatment*Season + (1|Round)	6	−78.60	172.6	0.43	0.24
Treatment + Season + (1|Round)	5	−80.35	173.0	0.86	0.20
Treatment + Habitat + (1|Round)	5	−81.14	174.6	2.45	0.09
Treatment*Season + Habitat + (1|Round)	7	−78.38	175.4	3.29	0.06
Treatment + Habitat + Season + (1|Round)	6	−80.15	175.7	3.52	0.05
Treatment*Habitat + (1|Round)	6	−80.48	176.3	4.19	0.04
Treatment*Habitat + Season + (1|Round)	7	−79.45	177.6	5.42	0.02
Null Model + (1|Round)	3	−88.89	184.6	12.50	0.00
Season + (1|Round)	4	−88.28	186.0	13.91	0.00
Habitat + (1|Round)	4	−88.77	187.0	14.89	0.00

*Note:* Predictor variables and deployment ‘round’ as a random effect (Model), number of parameters (*k*), log‐likelihood (Log*L*), Akaike's Information Criterion with small‐sample bias adjustment (AICc), ΔAICc (ΔAIC = AIC_*i*_ − AIC_min_) and model weights (*w*_*i*_).

### 3.2 Experimental Vulture Exclusion Effect on Fly Abundance

**FIGURE 3:**
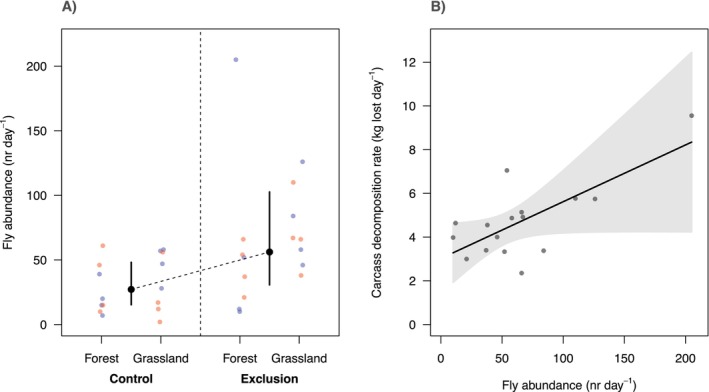
Effects of vulture exclusion on fly abundance (nr day^−1^) (A) and effect of fly abundance on carcass decomposition rates (kg lost day^−1^) at exclusion sites (B). In panel (A), black points represent the predicted fly abundance from the best supported model in Table 2; vertical bars = 95% confidence interval for the fixed effect predictions; coloured dots represent the raw data (red = dry season; blue = wet season). In panel B, black line = predicted change in decomposition rate with increasing fly abundance at a given exclusion site; grey polygon represents the 95% confidence interval around the fixed effects; and grey dots represent the raw data (exclusion sites only).

There was strong statistical support for the influence of treatment on the abundance of flies at carcasses (Table 2; Figure 3A), whereby predicted fly abundance at exclusion sites (mean = 56.1 flies day^−1^, CI = 30.7–103) was approximately double that predicted at control sites (mean = 29.9 flies day^−1^, CI = 20.3–44.3). The best supported model contained only the treatment effect (ΔAICc = −5.43 relative to the null model; Table 2) and explained 33.7% of the variation in fly abundance. There was weaker support for habitat and no support for season or the interaction between habitat and treatment predicting fly abundance (Table 2; i.e., models containing these variables in addition to treatment scored weaker AICcs than the model with treatment alone). There was strong statistical support for fly abundance predicting the carcass decomposition rate in the vulture exclusion treatments (ΔAICc = −8.64 relative to the null model), whereby the predicted decomposition rate at the lowest recorded fly abundance was 3.3 kg lost day^−1^ (CI = 1.9–4.6), whereas it was 8.3 kg lost day^−1^ (CI = 4.2–12.5) at the highest recorded fly abundance (Figure 3B).

**TABLE 2:** Model selection results explaining fly abundance for 32 pig carcasses from which vultures were experimentally excluded or not (treatment) across two habitats (grassland and forest) and seasons (dry and wet).

Model	*k*	Log*L*	AICc	ΔAICc	*w*_*i*_
**Treatment + (1|Round)**	**4**	**−150.10**	**309.7**	**0.00**	**0.39**
Treatment + Habitat + (1|Round)	5	−149.05	310.4	0.71	0.27
Treatment + Season + (1|Round)	5	−149.92	312.1	2.45	0.11
Treatment + Habitat + Season + (1|Round)	6	−148.96	313.3	3.60	0.06
Treatment*Habitat + (1|Round)	6	−149.00	313.4	3.68	0.06
Null Model + (1|Round)	3	−154.13	315.1	5.43	0.03
Treatment*Season + (1|Round)	6	−149.91	315.2	5.49	0.03
Habitat + (1|Round)	4	−153.23	315.9	6.26	0.02
Treatment*Habitat + Season + (1|Round)	7	−148.91	316.5	6.80	0.01
Treatment*Season + Habitat + (1|Round)	7	−148.96	316.6	6.90	0.01
Season + (1|Round)	4	−153.99	317.5	7.77	0.01

*Note:* Fixed effect predictor variables and deployment ‘round’ as a random effect (Model), number of parameters (*k*), log‐likelihood (Log*L*), Akaike's information Criterion with small‐sample bias adjustment (AICc), ΔAICc (ΔAIC = AIC_*i*_ − AIC_min_) and model weights (*w*_*i*_).

### 3.3 Experimental Vulture Exclusion Effect on Dung Beetle Abundance

**FIGURE 4:**
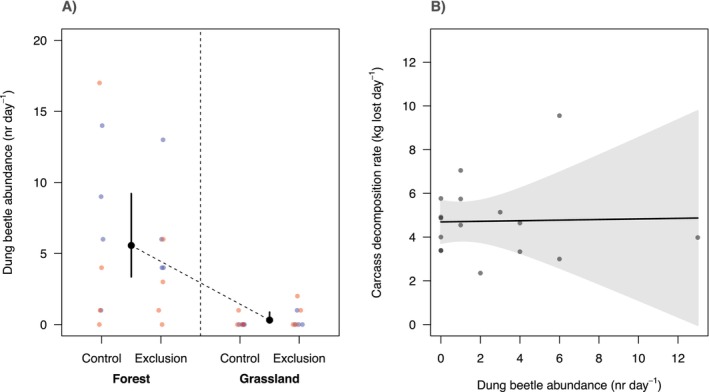
Effects of vulture exclusion on dung beetle abundance (nr day^−1^) (A) and effect of dung beetle abundance on carcass decomposition rates (kg lost day^−1^) at exclusion sites (B). In panel A, black points represent the predicted dung beetle abundance from the best supported model in Table 3; vertical bars = 95% confidence interval for the fixed effect predictions; coloured dots represent the raw data (red = dry season; blue = wet season). In panel B, black line = predicted change in decomposition rate with increasing dung beetle abundance at a given exclusion site; gry polygon represents the 95% confidence interval around the fixed effects; and grey dots represent the raw data (exclusion sites only).

There was strong statistical support for habitat predicting the abundance of dung beetles at carcasses (Table 3; Figure 4A), whereby predicted dung beetle abundance at forest sites (mean = 5.6 dung beetles day^−1^, CI = 3.4–9) was markedly higher than that predicted at grassland sites (mean = 0.3 dung beetles day^−1^, CI = 0.1–0.9). The best‐supported model contained only the habitat effect (ΔAICc = −19.84 relative to the null model; Table 3) and explained 65.4% of the variation in dung beetle abundance. There was no support for treatment, season or the interaction between habitat and treatment predicting dung beetle abundance (Table 3; i.e., models containing these variables in addition to habitat scored appreciably weaker AICcs than the model with habitat alone). Furthermore, there was no statistical support for species‐specific responses to the vulture exclosure when considered individually (Tables S1 and S2 in Appendix S1). There was also no statistical support for dung beetle abundance predicting carcass decomposition rate (Figure 4B) within the vulture exclusion treatments (ΔAICc = +3.07 relative to the null model).

**TABLE 3:** Model selection results explaining dung beetle abundance for 32 pig carcasses from which vultures were experimentally excluded or not (treatment) across two habitats (grassland and forest) and seasons (dry and wet).

Model	*k*	Log*L*	AICc	ΔAICc	*w*_*i*_
**Habitat + (1|Round)**	**4**	**−56.20**	**121.9**	**0.00**	**0.67**
Treatment + Habitat + (1|Round)	5	−56.20	124.7	2.82	0.16
Treatment*Habitat + (1|Round)	6	−55.17	125.7	3.81	0.10
Treatment + Habitat + Season + (1|Round)	6	−56.06	127.5	5.58	0.04
Treatment*Habitat + Season + (1|Round)	7	−54.95	128.6	6.68	0.02
Treatment*Season + Habitat + (1|Round)	7	−56.01	130.7	8.80	0.01
Null model + (1|Round)	3	−67.43	141.7	19.84	0.00
Season + (1|Round)	4	−67.12	143.7	21.84	0.00
Treatment + (1|Round)	4	−67.34	144.2	22.28	0.00
Treatment + Season + (1|Round)	5	−66.99	146.3	24.40	0.00
Treatment*Season + (1|Round)	6	−66.90	149.2	27.27	0.00

*Note:* Predictor variables and deployment ‘round’ as a random effect (Model), number of parameters (*k*), log‐likelihood (Log*L*), Akaike's Information Criterion with small‐sample bias adjustment (AICc), ΔAICc (ΔAIC = AC_*i*_ − AIC_min_) and model weights (*w*_*i*_).

## 4 Discussion

We experimentally tested the effect of vulture exclusion on carcass decomposition rate and fly and dung beetle abundance within two different habitats (grassland and secondary growth forest) and seasons (wet and dry) in the Neotropics. The experimental exclusion of vultures from carcasses approximately halved carcass decomposition rate, and natural variation in vulture abundance at control carcasses positively predicted carcass decomposition rate. Both findings support vultures playing a critical role in carcass decomposition in the Neotropics. Vulture exclusion from carcasses also approximately doubled fly abundance, while leaving dung beetle abundance unaffected. We discuss the implications of our findings in terms of the ecological role of vultures in this ecosystem and the wider potential consequences of vulture population declines.

### 4.1 Vulture Exclusion Significantly Decreases Carcass Decomposition Rate

Our results demonstrate that intact scavenger communities, with vultures, invertebrate and microbial decomposers, efficiently decompose carcasses at double the rate of insect scavengers and microbial decomposers alone. Although other facultative vertebrate scavengers were also excluded from the treatment carcasses, their minimal presence at control carcasses (see Section 4.3) confirmed that vultures are likely the main drivers of carcass decomposition rate within this ecosystem. This corroborates evidence from studies in other regions and climates demonstrating slower decomposition rates and/or longer carcass persistence times when vultures are absent. For example, in Kenya, vulture exclusion led to carcasses persisting nearly three times longer, even though the number of mammalian scavengers at carcasses tripled (Ogada, Keesing, et al. 2012; Ogada, Torchin, et al. 2012). Similarly, in the United States, rabbit carcasses inaccessible to vultures but available to other vertebrate scavengers remained unscavenged 10 times more often than those accessible to vultures (Hill et al. 2018). And in northern Spain, carcass consumption rate was 125 times lower, with carcasses persisting approximately 12 days longer, when the scavenger assemblage did not contain griffon vultures compared to when they did (Oliva‐Vidal, Sebastián‐González, et al. 2022). Correlative findings comparing decomposition patterns in regions with and without vultures show similar patterns. For example, in a study by Morales‐Reyes et al. (2017), carcass consumption rate was 13‐fold lower in regions where vultures were absent compared to regions where they were present. Our findings also indicate that the relative vulture abundance at carcasses is positively correlated with carcass decomposition rate, suggesting that a healthy population density is essential for maintaining high rates of carcass removal.

We also assessed the role of two further factors thought to influence decomposition rates, habitat and season. Since two of the three vulture species in this landscape rely on sight to find carrion (black and king vulture), we initially expected that control carcasses in grasslands, being more visible and thus more detectable, would be scavenged faster than those under canopy (Houston 1985; Oliva‐Vidal, Sebastián‐González, et al. 2022). Interestingly, we found no interaction between habitat type and treatment, likely due to vultures discovering all forest control carcasses. This is in contrast to the aforementioned study by Oliva‐Vidal, Sebastián‐González, et al. (2022) in Europe, where carcass detection by griffon vultures markedly decreased in forests compared to open landscapes. We attribute our results to the presence of the turkey vulture in this ecosystem, the only vulture species known to locate carrion by scent due to its highly developed olfactory bulb (Grigg et al. 2017). It has been suggested that other vulture species, such as the king vulture, follow turkey vultures into the forest after the latter have located carrion (Houston 1984). Accordingly, we detected that turkey vultures were the first to arrive at six of the eight forest control carcasses, with black and king vultures arriving later. This could explain why control carcass decomposition rates do not vary between habitats.

We also expected increased decomposition rates in control carcasses during the dry season as vultures fly more during warm and clear weather to take advantage of thermals, increasing their encounter probability with carcasses (Bohrer et al. 2012; Dodge et al. 2014). However, there was no statistical support for a difference in control carcass decomposition rates between seasons. This further strengthens the possibility that socially acquired information plays an important role in carcass discovery (Bildstein 2022). For example, griffon vultures improve their chances of finding carcasses by detecting when other vultures land at carrion sites and following them (Cortés‐Avizanda et al. 2014). We did find some support for carcass decomposition rates being faster in the wet season than the dry season under the vulture exclusion treatment (support for a treatment by season interaction). This slight increase in decomposition rates for carcasses inaccessible to vultures in the wet season may be due to increased rainfall. This can alter temperature and humidity, both of which have complex interactive effects on the carcass itself and its decomposition by both insects and microbes (Payne 1965; DeVault et al. 2004; Benbow et al. 2013; Pechal et al. 2013). Interestingly, the El Niño‐Southern Oscillation event (a weather system that typically makes the wet seasons drier than usual; see Esquivel‐Hernández et al. 2019) occurred during the year in which we conducted the wet season rounds. The difference in the effect of vulture exclusion on carcass decomposition rate between seasons may therefore be more pronounced in years without El Niño. Since decomposition rates in control carcasses did not vary between seasons, a balanced scavenger community of vertebrates and invertebrates may help stabilize these seasonal effects on carcass decomposition.

### 4.2 Invertebrate Abundance Responses to Vulture Exclusion

Carrion is a finite and ephemeral resource generating intense competition among insects, leading to density‐dependent population dynamics and increased contact between potential zoonosis vectors (DeVault et al. 2003; Blackburn et al. 2014; Dawson et al. 2022; Patel et al. 2022). We found that vulture exclusion and the related decrease in decomposition rate resulted in taxon‐specific responses in insect abundance. Our estimates of daily fly abundance approximately doubled when vultures were excluded. This likely resulted from fly abundance and reproductive success being dependent upon carcass longevity, with more adult flies arriving over time and larvae surviving to adulthood when sufficient food and breeding resources are available (Rivers et al. 2011; Barton and Evans 2017). Flies maximise carcass utilisation by laying hundreds to thousands of eggs, which develop rapidly into larvae forming intensively feeding maggot masses that eventually mature into adult flies (Rivers et al. 2011). This process was likely hindered by vulture carrion consumption at the control sites, preventing more adult flies from finding the carcass and larvae from reaching adulthood.

However, there was no statistical support for a change in dung beetle abundance with vulture exclusion. While flies utilise carrion throughout all stages of their life cycle, adult dung beetles rely on carrion primarily for feeding and breeding (Hanski and Cambefort 1991; Halffter et al. 2011). The life cycle of dung beetles is also much slower than that of flies, with females laying from 1 to 10 eggs per nest, which may require up to 2 months to mature into adulthood (Bennett and Whitworth 1991; Huerta et al. 2023). Therefore, dung beetle abundance at a carcass likely depends on the existing adult population in the surrounding area and not their progeny. Consequently, carcass longevity may not influence dung beetle abundance if it is determined by the local adult beetle population.

There was no strong support for fly and dung beetle abundance varying with season, perhaps as insects tend to proliferate in either hot or humid climates, making both seasons in the tropics optimal for insect activity (Payne 1965). However, we did find strong statistical support for dung beetle abundance varying by habitat, with dung beetles being more abundant in forest relative to grassland. This is consistent with previous work from the same region which found increased dung beetle abundance and diversity in forest compared to grassland (Whitworth et al. 2021, see also Table S2 in the Appendix S1).

Vultures are known to also consume insects, which could exert a control on their population (e.g., Hill et al. 2022). If vultures preyed on flies at control sites, this may have influenced our results, leading to reduced fly abundance at control carcasses compared to exclusion carcasses. Likewise, carcasses left unscavenged by vertebrates may also attract more predatory insects over time, which in turn could control fly and dung beetle populations (Dawson et al. 2022). Future experiments examining the responses of invertebrates to vulture exclusion in different ecoregions are required to verify the generalities of these findings.

### 4.3 Do Carcasses Support a Broad Facultative Scavenger Community?

Studies in temperate regions often find that animal carcasses can support a diverse facultative scavenger community (e.g., Hill et al. 2018; Tobajas et al. 2021; Oliva‐Vidal, Sebastián‐González, et al. 2022). However, this did not appear to be the case in this Neotropical forest/agricultural matrix system. Whilst we expected that coatis, opossums, pumas, ocelots and generalist birds would opportunistically scavenge in this ecosystem, they were rarely observed interacting with the control carcasses. Camera trap photos showed that vultures were the primary vertebrate drivers of decomposition rate at carcasses available to them as they consumed all but one control carcass. During the first 10 days of the carcass deployment, the only vertebrates other than vultures recorded at control carcasses were opossums (12 of 16 deployments) and a white‐nosed coati (1 of 16). Common opossums were observed at almost all control carcasses and managed to breach five exclusion cages. Given that opossums and coatis are omnivorous, and we did not observe direct scavenging of the carcass, it is also possible that both species consumed only insects at the sites (Gompper 1995; Lessa et al. 2022). The relative lack of facultative scavenging in this system may be due to competitive exclusion by vultures at carcasses (Butler and du Toit 2002; Ogada, Keesing, et al. 2012; Ogada, Torchin, et al. 2012). Previous work has highlighted that both vertebrate and invertebrate scavenger abundance and community composition are influenced by carcass size, potentially affecting carcass decomposition rate (DeVault et al. 2004; Tomberlin et al. 2017; Turner et al. 2017; Oliva‐Vidal, Sebastián‐González, et al. 2022). As we only used large carcasses > 35 kg, it is possible that facultative scavenging occurs more frequently on small to medium‐sized carcasses. Experiments using carcasses of varying sizes could shed light on the interaction between obligate and facultative scavengers, and whether invertebrates are more efficient than vultures at removing smaller carcasses (Moretti et al. 2008; Sugiura and Hayashi 2018; Oliva‐Vidal, Sebastián‐González, et al. 2022).

### 4.4 Implications of Vulture Decline for Carcass Decomposition

Vulture population declines in this region will likely lead to increased carcass persistence since facultative scavengers seem rare in this neotropical system. One implication of such a change would be that nutrients from carrion take longer to return to the environment (DeVault et al. 2003) and would stay highly localised instead of being distributed across the landscape (through vulture scat deposition). Previous work on mass vertebrate mortality events demonstrated that the local concentration of phosphorous, sodium, potassium and nitrogen from carrion can alter the growth of surrounding vegetation (Barton et al. 2013; Tomberlin et al. 2017).

Slower carcass decomposition rate may also have important consequences for infectious and zoonotic disease management in the tropics. For example, Aguilar‐Vargas et al. (2022) found that 27% of 85 wildlife carcasses brought in for epidemiological surveillance in Costa Rica belonged to animals that had died from an infectious pathogen. Vultures could help mitigate the spread of these pathogens (Ogada, Keesing, et al. 2012; Ogada, Torchin, et al. 2012; Roggenbuck et al. 2014) particularly as microbes rarely survive the passage through a vulture's digestive system (Houston and Cooper 1975). Nonetheless, some authors have suggested that vultures could also spread zoonoses as they move among carcasses and drinking sites (e.g., Houston and Cooper 1975; Houston 1979; DeVault et al. 2004). However, to date, there has been no direct evidence of vultures transmitting or spreading disease. The implementation of low‐cost epidemiological surveillance systems such as Wildlife Health Monitoring Programmes could aid in the early detection of potential emerging zoonoses in and/or on vultures and other scavengers (Aguilar‐Vargas et al. 2022).

In contrast to vultures, both flies and dung beetles are known vectors of zoonoses. Dung beetles are carriers of enteric parasites such as the tapeworm (*Taenia hydatigena*), yet studies on their transmission abilities remain inconclusive (e.g., Nichols et al. 2008; Patel et al. 2022). Blowflies, usually the first species to colonise a carcass, are known vectors of both botulism and anthrax (e.g., Anza et al. 2014; Blackburn et al. 2014). Other microorganisms pathogenic to humans found in fly species include antibiotic‐resistant strains of *Escherichia coli* (Monyama et al. 2022), Salmonella spp. (Olsen and Hammack 2000) and Shigella spp., the main cause of dysentery (Mohammed et al. 2016). The increase in fly abundance observed at carcasses excluded from vultures in this experiment therefore raises concerns over the potential increased risk of disease transmission in areas experiencing vulture declines. Research on insect community composition at carrion in the Neotropics might therefore be usefully prioritised, with a view to identifying potential disease vectors and the transmission risk they pose. In regions facing vulture decline, testing the effect of facultative scavengers on carrion insects and carcass decomposition may also reveal potential new transmission pathways for emerging zoonoses.

## 5 Conclusions

This work presents the first experimental assessment of the implications of vulture declines on carcass decomposition in neotropical ecosystems. Our results add to a growing body of evidence that undisturbed scavenger communities containing vultures decompose carrion more efficiently than those without vultures (Ogada, Keesing, et al. 2012; Ogada, Torchin, et al. 2012; Morales‐Reyes et al. 2017; Hill et al. 2018; Oliva‐Vidal, Sebastián‐González, et al. 2022). While it would be beneficial to replicate this work in different neotropical ecosystems (e.g., savannah) and contexts (e.g., urban environments) to determine how generalisable these results are, our findings do suggest that future declines in neotropical vulture populations will compromise ecosystem services associated with fast carcass decomposition.

## Author Contributions

**Julia Grootaers:** conceptualization (lead), data curation (lead), formal analysis (lead), investigation (lead), methodology (equal), project administration (equal), resources (lead), software (lead), visualization (lead), writing – original draft (lead), writing – review and editing (lead). **Greta Hernández Campos:** data curation (equal), investigation (equal), methodology (lead), project administration (equal), resources (equal), writing – review and editing (supporting). **Violeta Marie Montenegro:** data curation (equal), formal analysis (supporting), investigation (equal), methodology (equal), software (supporting), writing – review and editing (supporting). **Rosio Vega Quispe:** data curation (equal), investigation (equal), methodology (equal), writing – review and editing (supporting). **Sarah Wicks:** funding acquisition (equal), investigation (equal), project administration (equal), resources (equal), writing – review and editing (supporting). **Sara Campos Landázuri:** investigation (equal), methodology (equal), writing – review and editing (supporting). **Eduardo Fabrizio Tubelli:** investigation (equal), methodology (equal), writing – review and editing (supporting). **Francisco Vega‐Reyes:** investigation (equal), methodology (equal), writing – review and editing (equal). **Enzo Basso:** formal analysis (supporting), investigation (equal), methodology (equal), resources (equal), writing – review and editing (equal). **Andrew Whitworth:** conceptualization (lead), funding acquisition (lead), writing – review and editing (equal). **Andrew Young:** conceptualization (lead), methodology (lead), supervision (lead), writing – review and editing (lead). **Christopher Beirne:** conceptualization (lead), data curation (supporting), formal analysis (lead), investigation (supporting), methodology (lead), software (equal), supervision (lead), visualization (lead), writing – review and editing (lead).

## Ethics Statement

Pig carcasses were obtained from local farms with production for human consumption. Research permits were obtained from the Government of Costa Rica and CONAGEBIO (permit ref.: R‐CM‐ACO‐002‐2024‐OT‐CONAGEBIO). Our study did not involve direct management of living vertebrates; thus, no further ethical approval was required in accordance with current regulations on animal experimentation in the UK and Costa Rica (Animals Scientific Procedures Act 1986 (ASPA)).

## Conflicts of Interest

The authors declare no conflicts of interest.

## Supporting information


Appendix S1.

## Data Availability

The data and code that support the findings of this study are openly available in Zenodo at DOI: https://doi.org/10.5281/zenodo.15217934.
