# Supplementary material for: Vulture Exclusion Halves Large Carcass Decomposition Rates and Doubles Fly Abundance
Source: Ecol Evol. 2025 May 8;15(5):e71408. doi: 10.1002/ece3.71408 (PMC12061450; doi:10.1002/ece3.71408)
Supplement: Supplementary file 1 — Appendix S1. [file ECE3-15-e71408-s001.docx]

Appendix

**TABLE S1.** Dung beetle species present in treatments (exclusion and control) and habitats (grassland and forest).

| **Species** | **Treatment** | | **Habitat** | | **Total** |
| --- | --- | --- | --- | --- | --- |
|  | Exclusion | Control | Forest | Grassland |  |
| *Canthidium centrale* | 1 | 0 | 1 | 0 | 1 |
| *Canthon mutabilis* | 54 | 16 | 0 | 70 | 70 |
| *Copris brevicornis* | 2 | 0 | 1 | 1 | 2 |
| *Coprophanaeus corythus* | 58 | 53 | 108 | 3 | 111 |
| *Coprophanaeus pecki* | 27 | 11 | 38 | 0 | 38 |
| *Coprophanaeus solisi* | 8 | 1 | 7 | 2 | 9 |
| *Deltochilium gibbosum* | 1 | 2 | 3 | 0 | 3 |
| *Deltochilium pseudoparile* | 10 | 31 | 41 | 0 | 41 |
| *Eurysternus foedus* | 12 | 14 | 25 | 1 | 26 |
| *Eurysternus plebejus* | 3 | 4 | 7 | 0 | 7 |
| *Onthophagus acuminatus* | 340 | 239 | 563 | 16 | 579 |
| *Onthophagus coriaceoumbrosus* | 18 | 7 | 25 | 0 | 25 |
| *Onthophagus marginicollis* | 0 | 1 | 0 | 1 | 1 |
| *Onthophagus praecellens* | 133 | 177 | 309 | 1 | 310 |
| *Onthophagus sharpi* | 0 | 1 | 1 | 0 | 1 |
| *Pedaridium pilosum* | 1 | 0 | 1 | 0 | 1 |
| *Scybalocanthon moniliatus* | 2 | 3 | 5 | 0 | 5 |
| *Sylvicanthon aequinoctialis* | 2 | 13 | 15 | 0 | 15 |

**TABLE S2.** We screened the potential for species-specific responses to the vulture exclosure treatment using the ‘mvabund’ package in R. We assessed the significance of two predictors (habitat and treatment) and the interaction between them using a significance threshold set as adjusted p < 0.05 after correcting p-values for multiple comparisons using a step-down resampling procedure. Overall, the multivariate effect of habitat was strongly significant (P = 0.001), whereas there was no statistical support for treatment (P = 0.637) or the interaction between habitat and treatment (P = 0.169). The following table details the univariate species-specific tests where: ‘Interaction’ relates to the interaction between habitat and treatment; values in the cells = the corrected p-values for each predictor (columns); values in bold and grey cells = significant predictors of species-specific dung beetle abundance.

| **Species** | **Habitat** | **Treatment** | **Interaction** |
| --- | --- | --- | --- |
| *Canthidium centrale* | 0.908 | 0.974 | 0.981 |
| *Canthon mutabilis* | **0.032** | 0.991 | 0.981 |
| *Copris brevicornis* | 0.908 | 0.828 | 0.981 |
| *Coprophanaeus corythus* | **0.002** | 0.991 | 0.410 |
| *Coprophanaeus pecki* | **0.001** | 0.962 | 0.981 |
| *Coprophanaeus solisi* | 0.908 | 0.961 | 0.410 |
| *Deltochilium gibbosum* | 0.173 | 0.991 | 0.981 |
| *Deltochilium pseudoparile* | **0.015** | 0.984 | 0.981 |
| *Eurysternus foedus* | **0.004** | 0.992 | 0.410 |
| *Eurysternus plebejus* | **0.015** | 0.991 | 0.981 |
| *Onthophagus acuminatus* | **0.001** | 0.961 | 0.410 |
| *Onthophagus coriaceoumbrosus* | 0.082 | 0.991 | 0.981 |
| *Onthophagus marginicollis* | 0.891 | 0.987 | 0.981 |
| *Onthophagus praecellens* | **0.001** | 0.992 | 0.410 |
| *Onthophagus sharpi* | 0.908 | 0.974 | 0.981 |
| *Pedaridium pilosum* | 0.908 | 0.974 | 0.981 |
| *Scybalocanthon moniliatus* | **0.032** | 0.991 | 0.981 |
| *Sylvicanthon aequinoctialis* | 0.153 | 0.975 | 0.981 |
